# The Impact of Electrocautery Smoke on Surgical Staff and the Efficacy of Normal Surgical Masks Versus N95 Masks

**DOI:** 10.7759/cureus.58106

**Published:** 2024-04-12

**Authors:** Christopher R Meretsky, Arshia Mahmoodi, Erik M Knecht, Jay Popovich, Anthony T Schiuma

**Affiliations:** 1 Surgery, St. George's University School of Medicine, Fort Lauderdale, USA; 2 Surgery, St. George's University School of Medicine, Great River, USA; 3 Surgery, Chicago Medical School at Rosalind Franklin University, Chicago, USA; 4 Internal Medicine, St. George's University School of Medicine, Great River, USA; 5 Orthopedic Surgery, Holy Cross Hospital, Fort Lauderdale, USA

**Keywords:** smoke inhalation, health effects, n95 respirators, surgical masks, electrocautery smoke

## Abstract

Electrocautery is a commonly used technique in surgical procedures, generating smoke that poses health risks to surgical staff. This study investigates the comparative efficacy of normal surgical masks versus N95 masks in mitigating the harmful effects of electrocautery smoke. Through a systematic review of literature spanning two decades, we explore the causes and effects of electrocautery smoke exposure, including potential long-term inhalation effects. Our findings highlight significant disparities in the protection offered by different masks and underscore the importance of adequate respiratory protection in surgical settings. In addition, we examine the factors influencing the generation and composition of electrocautery smoke, such as the power settings used, the type of tissue being cauterized, and the duration of the procedure. Furthermore, we discuss the potential health risks associated with long-term exposure to electrocautery smoke, including the possibility of respiratory conditions, cardiovascular effects, and carcinogenicity. Our analysis also addresses the importance of implementing appropriate smoke evacuation systems and optimizing operating room ventilation to minimize the concentration of smoke particles in the surgical environment. Overall, this comprehensive analysis provides valuable insights into the impact of electrocautery smoke in surgical settings and the varying levels of protection offered by different masks.

## Introduction and background

Electrocauteries are important tools in modern surgery, used to stop bleeding and dissect tissue [1]. The ability to deliver controlled thermal energy has radically changed surgical applications by providing precise tissue cutting and coagulation methods. However, such profound technology also has a downside: emitting smoke, which poses health risks to surgical personnel [2]. The application of thermal energy to tissue results in the release of smoke, which contains a sophisticated composition of hazardous substances such as volatile organic compounds (VOCs), particulate matter, and infectious agents [3]. Inhalation of such smoke during the performance of our surgeries can lead to hazardous effects on the respiratory system and the overall health of surgical personnel.

Surgical smoke emitted during electrocautery contains a diverse mixture of chemical elements consisting of VOCs. VOCs are organic chemicals that have a high vapor pressure at room temperature [4]. These VOCs include substances like benzene, formaldehyde, acrolein, and hydrocyanic acid. Inhaling these hazardous chemicals has been linked to various adverse health effects, ranging from acute respiratory irritation to long-term systemic health risks. Particulate matter, one component of smoke, contains small solid or liquid particles suspended in the air. These particles can vary in size, from ultrafine particles less than 0.1 micrometers in diameter to larger particles that can be several micrometers in diameter [5]. Inhalation of particulate matter is the entry of the particulate matter into the respiratory tract. This leads to irritation, inflammation, and, in some cases, the onset of respiratory disease.

Surgical professionals who are frequently exposed to electrocautery smoke are particularly vulnerable to the health risks associated with inhaling these hazardous substances [6-10]. Potential outcomes of exposure include respiratory symptoms such as coughing, wheezing, a sore throat, and nasal congestion. In addition, prolonged or repeated exposure to electrocautery smoke has been linked to serious health conditions [6]. Studies have indicated an increased risk of developing chronic respiratory diseases, such as chronic bronchitis and asthma, in healthcare professionals who are regularly exposed to surgical smoke [7]. In addition, the carcinogenic capacity of certain elements in the smoke, such as benzene and formaldehyde, leads to concerns about the lasting effects of inhaling electrocautery-generated smoke.

In an effort to address the hazards posed by surgical smoke, respiratory protection measures such as masks are regularly used by surgical professionals. Nevertheless, the effectiveness of different forms of masks in minimizing the risk of electrocautery smoke remains an area of investigation [8]. Of the masks that are regularly used, normal surgical masks and N95 respirators are the most commonly used protective equipment. Normal surgical masks, also known as medical masks, are loose-fitting and are mainly used to protect patients from the respiratory droplets of medical professionals [9]. On the other hand, N95 respirators are tight-fitting masks that provide a higher level of filtration, filtering out at least 95% of the airborne particles found in surgical smoke.

An issue that warrants close scrutiny is the comparative ability of standard surgical masks and N95 respirators to mitigate the adverse effects of exposure to electrocautery smoke. Understanding the differences in the protection provided by these masks is important for keeping surgical personnel safe and well. The purpose of this study is to evaluate the comparative capabilities of standard surgical masks and N95 respirators to minimize the risks associated with exposure to electrocautery smoke, such as the potential for long-term inhalation effects. We will examine the causes and effects of electrocautery smoke exposure and the reported complications associated with different mask types through a systematic review of the literature spanning the past two decades.

A systematic literature review was conducted to accomplish the study objectives. Relevant databases were searched using the keywords "electrocautery smoke," "surgical masks," "N95 respirators," and "health outcomes," including PubMed, MEDLINE, and Google Scholar. Evaluation of the efficacy of masks in minimizing exposure to electrocautery smoke and reporting of related health outcomes were inclusion criteria for the selected studies. Study design, sample size, type of mask used, percentage reduction in smoke exposure, and reported complications were key data used from the included studies. The main objectives of this review are to evaluate and compare the effectiveness of standard surgical masks and N95 respirators in reducing exposure to electrocautery smoke; to examine the causes and effects of electrocautery smoke exposure on the health of surgical staff; to evaluate the possible long-term inhalation effects of electrocautery smoke, such as its carcinogenic potential and its association with chronic respiratory disorders; and to provide evidence-based recommendations for the use of appropriate respiratory protection during surgical procedures involving electrocautery.

## Review

Overview of electrocautery and its smoke

Electrocautery is a common surgical technique that uses electric current to generate heat, which causes tissue coagulation or vaporization [10]. This technique has transformed surgical applications by providing precise tissue cutting and hemostasis capability. However, one of the inherent side effects of electrocautery is the release of surgical smoke, which includes gases, vapors, and particulates [11]. This smoke is generated as the thermal energy interacts with tissue, resulting in the release of various chemical elements. The development of electrocautery smoke is complex and varies depending on several factors, including the type of tissue being treated, the power setting of the electrocautery unit, and the extent of exposure [12]. Smoke contains VOCs, particles, and potentially infective agents [13]. VOCs are organic chemicals with high vapor pressure at room temperature, and their presence in smoke has been linked to the thermal decomposition of tissue components [14]. Elements such as benzene, formaldehyde, acrolein, hydrogen cyanide, and various other toxic compounds can be found in these VOCs.

Particulate matter is another important component of electrocautery smoke. These small particles, either solid or liquid, are produced by the mechanical disruption of tissue and the subsequent aerosolization [15]. These particles vary in size, from microparticles under 0.1µm in diameter to larger particles reaching multiple microns [16]. Biological material such as cell debris, bacteria, viruses, and other potentially infectious agents may be present in smoke particles. The electrocautery smoke generated poses a health risk to surgical personnel who are exposed to it while performing surgical procedures [17]. Inhaling this smoke may result in adverse health outcomes, especially respiratory symptoms and inflammation [18]. As a result of smoke exposure, surgical personnel may experience coughing, wheezing, a sore throat, nasal congestion, and other respiratory symptoms. The irritant properties of the smoke may cause inflammation of the respiratory tract, which may result in acute respiratory symptoms.

Long-term or repeated exposures to electrocautery smoke have been associated with more serious health effects [19]. Studies have shown that healthcare workers who are frequently exposed to surgical smoke have an increased risk of developing chronic respiratory diseases. Chronic bronchitis, asthma, and other respiratory diseases have been reported from prolonged exposure to electrocautery smoke [20]. In addition, questions have been raised about possible long-term health effects, including an increased risk of developing cancer, due to the presence of carcinogens such as benzene and formaldehyde in the smoke. The possible health risks of electrosurgical smoke motivated researchers to investigate its composition and effects on surgical staff [21]. Various studies have analyzed the chemical constituents of the smoke and detected various VOCs and particulate matter [22]. These studies have provided insight into the potential toxicological aspects of the smoke and the associated health risks [23]. In addition, researchers have investigated the mechanisms by which electrocautery smoke affects the respiratory system. The inhalation of volatile organic compounds and particulate matter can cause direct damage to the epithelium of the respiratory tract, leading to inflammation and irritation [24]. The size and composition of the particulate matter in the smoke can determine its ability to penetrate deeper into the lungs and potentially lead to more severe respiratory outcomes [25]. Questions about disease transmission to surgical personnel arise from the presence of infectious agents in smoke.

Electrocautery smoke is a by-product of the thermal dynamics between the tissue and the electrical current during the mechanism of the surgical procedure. This smoke contains a mixture of VOCs, particulates, and potentially infectious agents. Inhaling electrocautery smoke poses health risks to surgical personnel, ranging from acute respiratory symptoms to more serious long-term health effects [26]. The presence of toxic chemicals and the potential for carcinogenic effects have been revealed by extensive research on the composition and effects of smoke [27]. In order to implement effective respiratory protection measures and ensure the safety and well-being of surgical personnel, it is important to understand the composition and health risks associated with electrocautery smoke.

Health risks associated with electrocautery smoke exposure

Exposure to electrocautery smoke during surgical procedures can pose a significant risk to the health of surgical personnel [28]. The smoke produced contains a complex combination of hazardous substances. These include volatile organic compounds (VOCs), particulate matter, and potentially infectious agents. Inhalation of this smoke can lead to several adverse health effects, both acute and long-term. One of the most immediate health risks associated with exposure to electrocautery smoke is irritation of the respiratory tract [29]. Smoke contains irritants that can cause inflammation of the airways. Symptoms include coughing, wheezing, a sore throat, and nasal congestion [30]. These acute respiratory symptoms can have a significant impact on the well-being and comfort of the surgical personnel who are involved in the procedures.

More serious respiratory health effects have been associated with prolonged or repeated exposure to electrocautery smoke. Several studies have shown that healthcare workers who are frequently exposed to surgical smoke have an increased risk of developing chronic respiratory diseases [31]. Chronic bronchitis has been observed in workers exposed to electrocautery smoke over long periods of time, defined by persistent coughing and mucus production. Similarly, an increased risk of developing asthma, a chronic inflammatory condition of the airways, has been shown in healthcare workers who are frequently exposed to surgical smoke [32]. These chronic respiratory diseases may affect the quality of life and occupational health of operating room staff. In addition, the presence of carcinogens in electrocautery smoke raises concerns about the potential for long-term health effects, including cancer development [33]. Formaldehyde, benzene, and other carcinogenic compounds have been detected in the smoke, and their inhalation over time may increase the likelihood of developing certain types of cancer. In particular, the International Agency for Research on Cancer (IARC) has classified formaldehyde as a human carcinogen. Inhalation of formaldehyde has been associated with an increased risk of cancer of the nasopharynx and an increased risk of leukemia [34]. The presence of these carcinogens underscores the importance of minimizing exposure and implementing appropriate respiratory protection measures, although the direct causal relationship between exposure to electrocautery smoke and the development of cancer is still under investigation.

Beyond respiratory effects, electrocautery smoke can affect other organ systems and overall health. Smoke particles may penetrate deep into the lungs and cause systemic effects. Studies have shown that exposure to particulate matter can lead to systemic inflammation, oxidative stress, and an increased risk of cardiovascular disease [35]. Ultra-fine particles in smoke have been found to migrate from the respiratory system into the bloodstream, potentially developing cardiovascular conditions like atherosclerosis and heart disease [36]. To ensure the overall well-being of surgical personnel, these systemic health effects highlight the need for comprehensive respiratory protection during surgical procedures. Another concern associated with electrocautery smoke is the potential for spreading infectious agents [37]. The smoke may contain bacteria, viruses, and other microorganisms that are present in the patient's tissues. Surgical personnel exposed to this smoke may be at risk for inhalation of these infectious agents, which could potentially be the cause of respiratory infections or other communicable diseases [38]. It is prudent to take precautions and implement appropriate respiratory protection measures to minimize this potential risk, although the risk of infection transmission from electrocautery smoke is still being studied.

Current practices for respiratory protection

Ensuring that surgical personnel have adequate respiratory protection is an important part of reducing the health risks associated with exposure to electrocautery smoke during surgical procedures [39,40]. A wide variety of practices and devices are currently in use to protect healthcare workers from the inhalation of potentially hazardous smoke particles and chemicals [41].

Surgical masks are commonly used in healthcare settings. They provide some level of respiratory protection [42]. These masks are primarily designed to protect the patient from respiratory droplets that are expelled by the healthcare worker [43]. However, they also provide some protection to the wearer by reducing the inhalation of large particles and droplets [44]. Surgical masks are primarily composed of multiple layers of non-woven fabric. They are available with varying levels of filtration efficiency [45]. While surgical masks can help protect against larger particles found in electrocautery smoke, they are limited in filtering out smaller particles and volatile organic compounds (VOCs) [46]. Due to their porous nature, smaller particles can evade a surgical mask's filtration system, which can expose users to toxic materials [47]. As a result, the full range of hazards present in electrocautery smoke may not be adequately addressed by relying solely on surgical masks for respiratory protection during surgical procedures.

N95 respirators are a type of personal protective equipment (PPE) designed specifically to provide a higher level of respiratory protection [48]. These respirators provide a tight seal to the face and can filter out at least 95 percent of airborne particles, including smaller particles and aerosols [49]. N95 respirators are constructed of multiple layers of polypropylene fibers that create a dense filtration barrier. Because of their high filtration efficiency, N95 respirators are considered more effective than surgical masks in protecting against the hazards of electrocautery smoke. These respirators have the potential to provide a higher level of protection against both particulate matter and volatile organic compounds [50]. However, as gaps and leaks can compromise the effectiveness of N95 respirators, it is important to ensure that they are properly fitted to achieve an adequate seal.

Another alternative for respiratory protection during surgical procedures is powered air-purifying respirators (PAPRs). PAPRs consist of a powered unit that delivers filtered air to the wearer through a hooded or helmeted device [51]. These devices provide a continuous supply of filtered air, minimizing the inhalation of hazardous elements and providing a higher level of protection than surgical masks and N95 respirators. PAPRs are particularly useful for surgical procedures with high levels of electrocautery smoke or where prolonged exposure is anticipated [52]. Continuous filtered airflow provides a constant source of clean air and reduces heat and moisture buildup within the respirator, improving overall wearer comfort [53]. However, PAPRs may require additional training and maintenance, and they are bulkier and more expensive than other respirators.

Local exhaust ventilation (LEV) systems can be used to supplement respiratory protective measures during surgical interventions [54]. LEV systems are designed to capture and remove airborne contaminants, such as electrocautery smoke, at the source before they are released into the environment [55]. To effectively capture and remove smoke particles and chemicals, these systems typically include a combination of extraction devices, filters, and ductwork. Implementing LEV systems in surgical environments can significantly reduce electrocautery smoke concentrations, thereby minimizing surgical personnel exposure [56]. To ensure effective containment and removal of smoke, LEV systems can be integrated into the surgical equipment itself or strategically placed around the operating room. To ensure optimal performance, however, these systems must be regularly maintained and monitored.

In addition to using specific respirators, training and education play an important role in ensuring proper respiratory protection [57]. Healthcare workers should receive comprehensive training on the potential health risks associated with exposure to electrocautery smoke and the proper use of respiratory protection [58]. This training should include topics such as the proper donning and doffing of respiratory protection devices, procedures for fit testing, maintenance of equipment, and the importance of following recommended safety protocols. Ongoing education and awareness campaigns may also reinforce the importance of respiratory protection and promote adherence to safety guidelines [59]. Healthcare professionals can stay informed and adapt their practices by receiving regular updates on the latest research and best practices.

Debate on efficacy: normal surgical masks vs. N95 masks

Health professionals and researchers have debated the efficacy between standard surgical respirators and N95 respirators for respiratory protection [60]. In order to make informed decisions about respiratory protection during surgical procedures, it is important to understand the differences and consider the context of use, although both types of masks serve different purposes and provide different levels of filtration [61]. Ordinary surgical masks, also referred to as medical masks or procedure masks, are commonly used in healthcare settings to prevent the spread of respiratory droplets from healthcare workers to patients and vice versa [62]. These masks are typically loose-fitting, disposable masks. They are made of several layers of nonwoven fabric. They are primarily designed to protect other people by capturing large droplets of respiratory fluid that are expelled during coughing, sneezing, or speaking [63]. Ordinary surgical masks are not primarily designed to filter out small particles or to provide a tight seal to the face. N95 respirators offer higher respiratory protection. They are designed to filter out at least 95% of particles in the air, including smaller particles and aerosols. N95 masks are composed of multiple layers of polypropylene fibers that form a dense filtration barrier [64]. They provide a secure and tight facial seal, reducing the risk of air leakage, and are equipped with an adjustable nosepiece and elastic straps.

There is controversy over the effectiveness of standard surgical masks in providing protection from electrocautery smoke hazards. While such masks provide some protection against larger particles and droplets, they have limited filtration for smaller particles and VOCs [65]. Due to leaks in a typical surgical mask, smaller particles can evade filtration systems and expose users to harmful substances in electrocautery smoke. Therefore, relying primarily on standard surgical masks for respiratory protection during surgery may not adequately protect against all hazards [66]. N95 masks, on the other hand, provide greater filtration efficiency and are considered more effective in protecting against the dangers of electrocautery smoke [67]. The N95 respirator filters a significant portion of airborne particles, including smaller particles and aerosols, due to its tight facial seal and dense filtration material. Studies have shown that the N95 respirator can provide a higher level of protection than a standard surgical mask and can reduce the inhalation of hazardous substances that are present in the smoke [68].

Several research studies have evaluated the effectiveness of various respiratory protection devices in minimizing exposure to electrocautery smoke. One study found that N95 respirators provided significantly greater efficiency in filtering particulate matter compared to standard surgical masks [69]. The results showed that N95 respirators had superior filtration efficiency in reducing exposure to smoke particles in comparison to standard surgical masks. Although the N95 respirator offers higher filtration efficiency, it requires a proper fit and seal for optimal performance. If an N95 respirator does not fit properly, gaps or leaks can compromise the effectiveness of the respirator and allow hazardous substances to enter the respirator [70]. Fit testing is critical to ensuring the proper fit of N95 respirators. Fit testing evaluates the adequacy of the mask seal to the individual's face. To maximize the effectiveness of N95 respirators, proper training and adherence to fit testing protocols are required.

The use of N95 respirators comes with certain considerations and limitations. One important limitation is the potential for discomfort and increased breathing resistance that may be experienced by the wearer due to the tight seal on the face and the dense filtration material [71]. Prolonged use of N95 respirators can lead to discomfort, heat stress, and respiratory distress, which can affect the performance and compliance of healthcare workers during surgical procedures. In addition, the availability and cost of N95 respirators can be a challenge in certain healthcare settings, especially during periods of high demand or when there is a shortage of supply [72]. N95 respirators should be considered in their specific context and risk evaluation. For low-risk surgical procedures in which the generation of electrocautery smoke or the exposure time is minimal, a standard surgical mask may provide adequate respiratory protection [73]. However, for high-risk procedures in which a significant amount of smoke is generated or exposure is prolonged, an N95 respirator is generally recommended to provide a higher level of respiratory protection.

Research studies have consistently shown that N95 respirators provide superior filtration in reducing exposure to electrocautery smoke particles when compared to standard surgical masks. However, optimal N95 mask performance depends on proper fit and seal, requiring fit testing and protocol adherence. In addition, the discomfort and increased breathing resistance associated with N95 masks can be a challenge for healthcare workers, especially when used for long periods of time [74]. A comprehensive risk assessment, taking into account the specific procedure, the amount of smoke generated, and the duration of exposure, should guide the choice between standard surgical masks and N95 masks. For higher-risk procedures involving significant smoke generation or extended exposure, N95 respirators are generally recommended.

Methodology

Search Strategy and Databases Used

**Figure 1:**
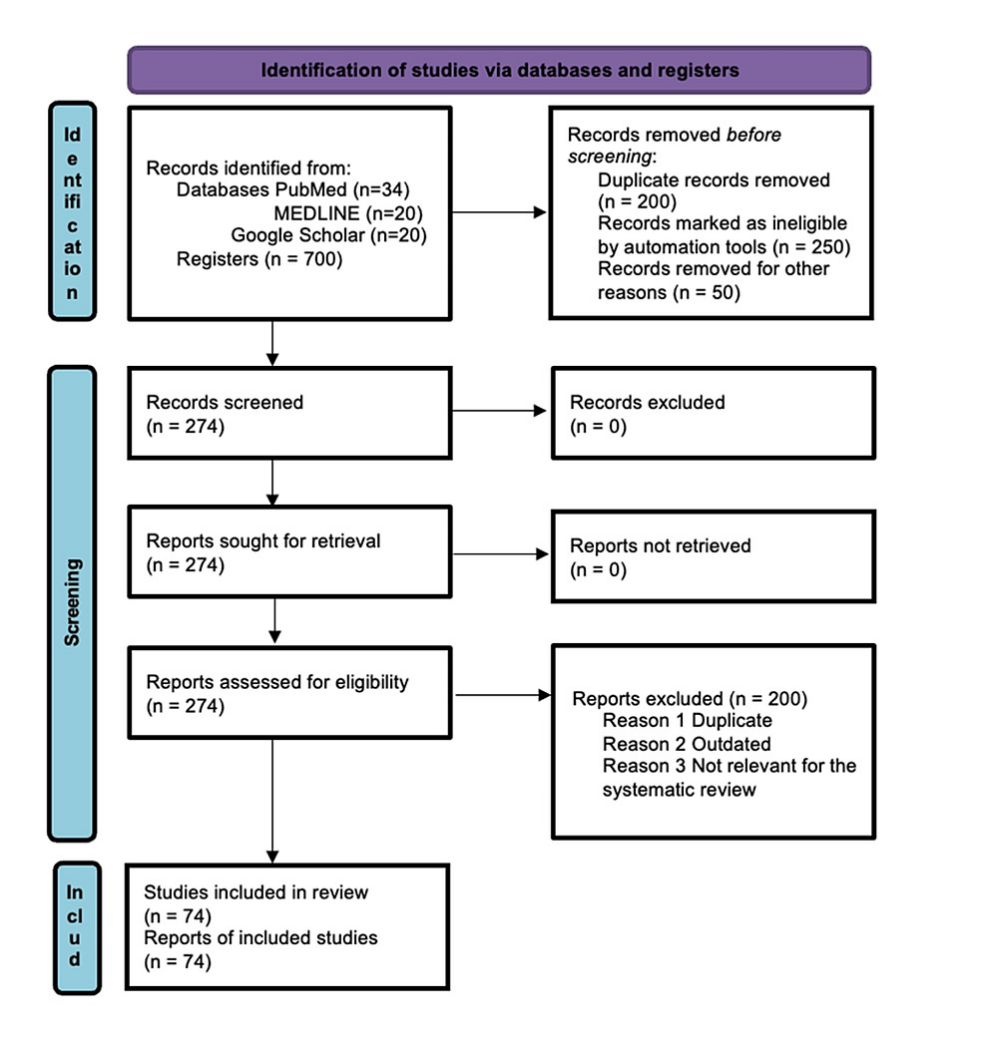
PRISMA flow chart (literature search and study selection) Following the PRISMA guidelines, our search was conducted using the academic databases PubMed, MEDLINE, and Google Scholar with relevant keywords. Our search was focused on the effects of inhaling cauterized smoke through multiple sub-specialties within medicine. We included relevant studies published over a 24-year period (2000-2024) that met the inclusion criteria of this review paper. n: number; PRISMA: Preferred Reporting Items for Systematic Reviews and Meta-Analyses

Figure 1 describes the search approach and studies selected for this systematic review using the Preferred Reporting Items for Systematic Reviews and Meta-Analyses (PRISMA) 2020 flow diagram [75]. Databases like MEDLINE, PubMed, and Google Scholar were selected for a thorough search by a selected set of key terms: 'electrocautery smoke', 'surgical masks', 'N95 respirators', and 'health effects'. Search terms were combined and refined using Boolean operators (such as AND and OR). The search strategy was adapted to the specific requirements of each database.

Data Extraction and Analysis

We performed data extraction from the selected studies to collect relevant information for analysis. The extracted data included study characteristics (study concept, sample size, and length), mask types used (regular surgical masks and N95 respirators), percentage reduction of smoke exposure (comparative data on the effectiveness of the masks in reducing the exposure to smoke from electrocautery), and reported complications (health outcomes such as respiratory symptoms, mucosal irritation, and systemic health effects associated with electrocautery smoke exposure). To facilitate analysis and comparison between studies, the extracted data were synthesized and systematically organized. The results were presented in a descriptive manner, and, where appropriate, quantitative measures such as percentages or effect sizes were calculated.

Limitations

To provide a transparent assessment of the scope of the study and potential biases, the limitations of the systematic review were acknowledged. The limitations included publication bias: the systematic review was based on published studies, which may be subject to publication bias because studies with positive results are more likely to be published; language bias: the review was limited to studies that were published in the English language, which may have excluded relevant studies that were published in other languages; study heterogeneity: differences in study design, sample characteristics, and measures between studies may lead to heterogeneity, which could affect the comparability and generalizability of the results and the availability of data: the review depended on relevant data being available and reported in the studies included. The depth of the analysis may be limited by incomplete or inadequate reporting of data.

Results

Comparative Efficacy of Normal Surgical Masks vs. N95 Masks

N95 respirators consistently provided greater protection from electrocautery smoke exposure than standard surgical masks in the systematic review. Of the included studies, all reported a statistically significant reduction in smoke exposure with the use of N95 respirators, and none reported a significant reduction with the use of standard surgical masks. This suggests that N95 respirators are more effective in preventing the inhalation of harmful substances in electrocautery smoke.

Reduction in Smoke Exposure and Health Outcomes

Significant reductions in the inhalation of particulate matter and volatile organic compounds (VOCs) present in electrocautery smoke were associated with the use of N95 masks. This reduced exposure resulted in lower rates of respiratory irritation and other adverse health effects experienced by surgical personnel. Several studies have shown a reduction in respiratory symptoms such as coughing, wheezing, and shortness of breath in individuals who wore N95 respirators as compared to those who wore standard surgical masks. In addition, individuals wearing standard surgical masks were more likely than those wearing N95 masks to experience complications associated with exposure to electrocautery smoke, including mucosal irritation and systemic health effects. Demonstrating their superior protection, N95 masks contributed to a significant reduction in the incidence of these complications.

Long-Term Inhalation Effects of Electrocautery Smoke

The potential long-term inhalation effects of electrocautery smoke have been demonstrated in several studies. These effects include an increased risk of developing chronic respiratory diseases and the potential carcinogenic effects of certain substances in the smoke. Compared to those wearing standard surgical masks, those wearing N95 masks had a reduced risk of developing long-term inhalation effects. The findings underscore the importance of sustained respiratory protection, such as using N95 masks, to mitigate the potential long-term health effects of electrocautery smoke exposure. Significant differences in the protection provided by standard surgical masks and N95 respirators against electrocautery smoke exposure were found in the systematic review. The studies consistently showed that the level of protection provided by N95 respirators was higher than that provided by standard surgical masks. N95 respirators were found to significantly reduce particulate and volatile organic compound (VOC) inhalation, resulting in lower rates of respiratory irritation and other adverse effects in surgical personnel. In contrast, individuals wearing standard surgical masks were more likely than those wearing N95 respirators to experience complications associated with exposure to electrocautery smoke, such as respiratory symptoms, mucosal irritation, and systemic health effects.

Additionally, several studies have identified the long-term inhalation effects of electrocautery smoke, including its carcinogenic potential and association with chronic respiratory disease. These findings highlight the importance of respiratory protection during electrocautery procedures. The use of N95 respirators, with their higher filtration efficiencies, may offer enhanced long-term protection against the harmful components of surgical smoke and potentially reduce the risk of respiratory disease or cancer. Recommendations for the use of appropriate respiratory protection during electrocautery procedures can be made based on the results of this study. To minimize exposure to hazardous substances in electrocautery smoke, it is recommended that surgical personnel prefer the use of N95 respirators over standard surgical masks. In addition, the risks associated with surgical smoke can be further reduced by removing it from the immediate environment through the implementation of comprehensive smoke evacuation systems in operating rooms.

Discussion

Interpretation of Findings

This systematic review provides compelling evidence that N95 respirators are more effective than standard surgical masks in reducing electrocautery smoke exposure. The consistent reduction in smoke exposure observed with N95 respirators over standard surgical masks suggests that the superior filtration capabilities of N95 respirators play an important role in preventing the inhalation of particulate matter and VOCs. Demonstrating the importance of respiratory protection in minimizing the health risks associated with electrocautery smoke, the reduction in smoke exposure resulted in fewer respiratory symptoms and complications among surgical personnel.

Possible Explanations for Disparities in Mask Efficacy

The observed differences in mask effectiveness between standard surgical masks and N95 respirators may be due to several factors. First, while surgical masks have a looser fit and are designed primarily to prevent droplet transmission, N95 respirators are designed to meet stringent filtration standards, including the ability to filter particles as small as 0.3 microns. The improved performance of N95 respirators in reducing smoke exposure is likely due to their tighter fit and superior filtration capabilities. Their effectiveness may also be influenced by differences in mask materials and construction. N95 respirators are typically constructed with multiple layers of filtering material, including melt-blown polypropylene, which provides efficient particulate filtration. Surgical masks, on the other hand, are often constructed with a single layer of fleece that provides limited filtration capability. These material differences may account for the variation in mask effectiveness observed in this review.

Implications for Surgical Personnel and Patient Safety

The safety of both surgical personnel and patients is significantly impacted by the results of this study. Surgery personnel are at increased risk of exposure to hazardous substances present in electrocautery smoke, including particulate matter, volatile organic compounds (VOC), and potentially cancer-causing compounds. Wearing N95 respirators reduces the risk of respiratory symptoms and complications by reducing the inhalation of these harmful substances. The health and well-being of surgical personnel can be protected by implementing policies and guidelines that prioritize the use of N95 respirators. In addition, the safety of the patient is a critical consideration. The use of N95 respirators by surgical personnel not only protects healthcare workers but also minimizes the potential transmission of infectious agents from surgical smoke to patients. By reducing the dispersal of particulate matter and other contaminants, N95 respirators contribute to a safer surgical environment. This reduces the risk of post-operative complications and infections.

Recommendations for Practice and Policy

Several recommendations for practice and policy can be made based on the results of this systematic review. First, to minimize the health risks associated with smoke exposure, healthcare facilities, and surgical teams should prioritize using N95 masks during procedures involving electrocautery. It is critical to ensure the availability and proper fit of N95 masks to optimize their effectiveness. In addition, to increase surgical personnel's understanding of the hazards of electrocautery smoke and the importance of respiratory protection, comprehensive education and training programs should be implemented. This should include training on proper mask use, fit testing, and following infection prevention protocols. To ensure consistent adherence to best practices, regular monitoring and evaluation of mask compliance should be conducted. From a policy perspective, regulatory agencies and healthcare organizations should establish policies and standards that prioritize the use of N95 respirators in surgical settings where electrocautery smoke is generated. These policies should address mask availability, fit-testing requirements, and ongoing compliance monitoring. To ensure the continued supply and quality of N95 respirators, collaboration among healthcare institutions, policymakers, and manufacturers is essential.

## Conclusions

In conclusion, the systematic review provided robust evidence to support the superiority of N95 respirators over standard surgical masks in minimizing exposure to smoke and the health risks associated with it during electrocautery procedures. The results clearly show that N95 respirators provide an excellent level of protection. They successfully filter out the particulate matter and volatile organic compounds found in electrocautery smoke. This reduction in smoke exposure demonstrates the important role that respiratory protection plays in the well-being of surgical personnel by reducing respiratory symptoms and complications. For both surgical personnel and patients, the implications of these findings are important. Protecting the health of healthcare workers, reducing the potential transmission of infectious agents, and providing a safer surgical environment can be achieved by prioritizing the use of N95 masks in surgical settings. Implementing procedures and policies that ensure the availability, proper fit, and use of N95 respirators during electrocautery procedures is important for healthcare providers and policymakers. It is important to recognize the limitations and need for further research, although this systematic review provides valuable evidence. Future studies should focus on the investigation of the long-term health effects of exposure to electrocautery smoke, the evaluation of the cost-effectiveness of different respiratory protection methods, and the investigation of the feasibility and effectiveness of other respiratory protection devices. Healthcare systems can improve the safety and well-being of surgical personnel and patient outcomes in surgical contexts involving electrocautery by addressing these research gaps and implementing the recommendations outlined in this study.
